# Odours of cancerous mouse congeners: detection and attractiveness

**DOI:** 10.1242/bio.059208

**Published:** 2022-04-29

**Authors:** Flora Gouzerh, Bruno Buatois, Maxime R. Hervé, Maicol Mancini, Antonio Maraver, Laurent Dormont, Frédéric Thomas, Guila Ganem

**Affiliations:** 1 Centre de Recherches Écologiques et Évolutives sur le Cancer, Maladies Infectieuses et Vecteurs: Ecologie, Genetique, Evolution et Contrôle, UMR IRD 224-CNRS 5290-Université de Montpellier, 34394, Montpellier, France; 2 Centre d'ecologie Fonctionnelle et Evolutive, Université de Montpellier, CNRS, EPHE, IRD, Université Paul Valéry Montpellier 3, 34293, Montpellier, France; 3 Institut de Génétique Environnement et Protection des Plantes, INRAE, Institut Agro, Université de Rennes, 35000, Rennes, France; 4 Institut de Recherche en Cancérologie de Montpellier, Inserm U1194-ICM-Université de Montpellier, 34298, Montpellier, France; 5 Institut des Sciences de l'Evolution, Université de Montpellier, CNRS, IRD, 34095, Montpellier, France

**Keywords:** Body odours, Odour discrimination, Female preference, Volatile organic compounds, Lung cancer, *Mus musculus domesticus*, EGFR oncogenic mutation

## Abstract

Chemical communication plays a major role in social interactions. Cancer, by inducing changes in body odours, may alter interactions between individuals. In the framework of research targeting non-invasive methods to detect early stages of cancer development, this study asked whether untrained mice could detect odour changes in cancerous congeners. If yes, were they able to detect cancer at an early developmental stage? Did it influence female preference? Did variations in volatile organic components of the odour source paralleled mice behavioural responses? We used transgenic mice strains developing or not lung cancer upon antibiotic ingestion. We sampled soiled bedding of cancerous mice (CC) and not cancerous mice (NC), at three experimental conditions: before (T0), early stage (T2) and late stage (T12) of cancer development. Habituation/generalisation and two-way preference tests were performed where soiled beddings of CC and NC mice were presented to wild-derived mice. The composition and relative concentration of volatile organic components (VOC) in the two stimuli types were analysed. Females did not show directional preference at any of the experimental conditions, suggesting that cancer did not influence their choice behaviour. Males did not discriminate between CC and NC stimuli at T0 but did so at T2 and T12, indicating that wild-derived mice could detect cancer at an early stage of development. Finally, although the VOC bouquet differed between CC and NC it did not seem to parallel the observed behavioural response suggesting that other types of odorant components might be involved in behavioural discrimination between CC and NC mice.

## Abstract (precis)

**Summary:** Male mice could discriminate the smell of cancerous congeners even when the tumour was hardly detectable by other means; however, females did not discriminate against the smell of males carrying cancerous tumours. Odorant molecules other than volatile organic compounds analysed here might explain the observed behaviour.

## INTRODUCTION

Odour based communication influences interspecies relationships and plays an essential role in the social life of many taxa (Caro and Balthazart, 2010; Mardon et al., 2010; Symonds and Elgar, 2008; Woodley, 2010; Wyatt, 2010). The information could be obtained via volatile (volatile organic compounds, VOCs) and non-volatile compounds (proteins, peptides, etc.). Both types of molecules contribute to olfactory communication in mammals. In general, long-distance communication requires volatile compounds while non-volatiles are involved in short-range communication and requires contact with the odour source (Muller-Schwarze, 2006). Odorant molecules are involved in signalling of, for example, species, sex, social rank, reproductive status and territory ownership (Ali et al., 2015; Célérier et al., 2010; 2011; Desjardins et al., 1973; Heth et al., 2001; Hurst and Beynon, 2004; Latour et al., 2014; Restrepo et al., 2006).

Olfaction, in addition to playing a key role in individual recognition for many species (Restrepo et al., 2006), also allows them to identify their congeners status in terms of stress and health. Such behaviour was observed in various vertebrate groups, such as fish, e.g. guppies *Poecilia reticulata* (Kennedy et al., 1987), and mammals including primates, e.g. *Mandrillus sphinx* (Poirotte et al., 2017), and rodents, e.g. *Cricetus cricetus* (O'Shea et al., 2002), or *Mus musculus domesticus* (Clayton, 1991; Ehman and Scott, 2001; Hurst, 2009; Kavaliers and Colwell, 1995). This ability allows individuals to reduce the risk of contagion, to avoid potentially costly aversive stimuli, or to ensure breeding with healthy partners (Thomas et al., 2005).

In view of the great olfactory capacity of some species, animals such as dogs or mice have been used as noses to detect various odour types. Dogs have been used in the detection of explosives and drugs (Gazit and Terkel, 2003; Jezierski et al., 2015), or human diseases such as epilepsy (Edwards et al., 2017; Strong and Brown, 2000) and more recently COVID-19 (Jendrny et al., 2020). Among rodents, the house mouse has been an emblematic model in such research (Clayton, 1991; Ehman and Scott, 2001; Ehmann et al., 2012; Su et al., 2018). Mice olfactory capacity was further demonstrated in studies showing their ability to distinguish between odours of kin of other species, e.g. Baboons *Papio ursinus* (Célérier et al., 2010) and blue petrels *Halobaena caerulea* (Célérier et al., 2011).

Like illnesses such as malaria (de Moraes et al., 2018), asthma (Ibrahim et al., 2011) or diabetes (Phillips et al., 2004), cancer can induce changes in individual odours (Buljubasic and Buchbauer, 2015; Sever et al., 2015). In recent years, animal noses have been used to detect early stages of cancer development and hence increase the effectiveness of treatments and chances of survival (Athey et al., 2012). Animals involved in such studies were dogs (Jezierski et al., 2015; Pirrone and Albertini, 2017), mice (Kokocinska-kusiak et al., 2020; Matsumura et al., 2010; Sato et al., 2017) and *Drosophila sp*. (Strauch et al., 2014).

As far as rodent cancers are concerned, research on how cancer may induce odour changes has identified the involvement of several VOC, such as p-cresol (Qiu et al., 2010), isoprene (Woollam et al., 2020), hexanal, hexane or benzaldehyde (Kokocinska-kusiak et al., 2020). Changes in relative concentration of mouse volatile pheromones were also reported, involving elevation of relative concentration of exo-brevicomin and 2-sec-butyl-4,5-dihydrothiazole in lung cancerous mice (Hanai et al., 2012). These molecules were shown to be present in higher relative concentrations in dominant as compared to subordinate males, and to induce female attraction (Hanai et al., 2012; Jemiolo et al., 1985).

In this study, we questioned whether cancer could alter female preference in mice and the ability of male to detect odour variation related to lung cancer at an early stage of development (i.e. not detected with IRM). As mentioned above, VOC were often pointed out as potential candidate molecules for cancer detection (Gouzerh et al., 2021), hence, we assessed whether changes in the mouse behaviour paralleled changes in the VOC composition of the stimuli presented to the mice during the behavioural tests. Specifically, we asked, (1) did the male's cancer status influence female preference for their odours? (2) Could untrained mouse detect the presence of cancer in odour sources of ill congeners? And if yes, were they able to detect cancer at an early developmental stage? (3) Did variations in VOC stimuli parallel mice behavioural responses to these stimuli?

## Results

### Female preference

**Fig. 1.**
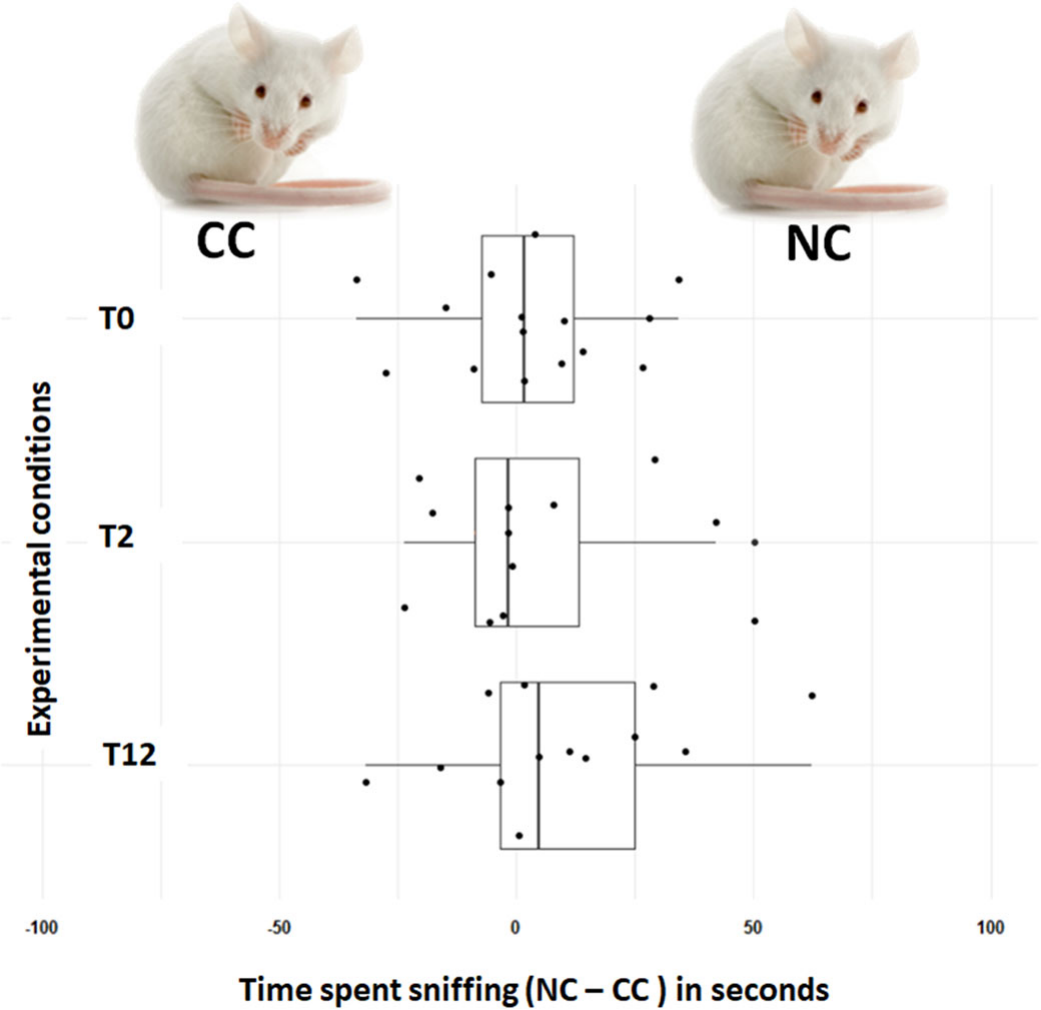
**Female preference for male stimuli.** A comparison of time spent sniffing the two stimuli (CC/NC), sampled at three experimental conditions (T0, T2, T12) during the two-way choice tests. Number of female mice tested in each trial is *n*=16 for T0, *n*=12 for T2 and *n*=13 for T12. A positive value indicates that a mouse spent more time sniffing stimulus NC, and a negative value indicates that a mouse spent more time sniffing the CC stimulus. Each dot is an individual measurement, and the boxplots picture the median (vertical line), the first and the third quartiles, and whiskers represent the 95% confidence interval.

We did not detect directional preference among wild-derived sexually receptive females at the three choice tests (T0: *N*=16, V=73, *P*-value=0.821, power=85%; T2: *N*=12, V=39, *P*-value=1, power=99%; T12: *N*=13, V=64, *P*-value=0.216, power=55%) (Fig. 1).

Moreover, duration of time spent smelling the two stimuli did not vary between experimental conditions [linear mixed model, (LMM) *F*_2,38=_0.873, *P*-value=0.426].

### Male discrimination

**Fig. 2.**
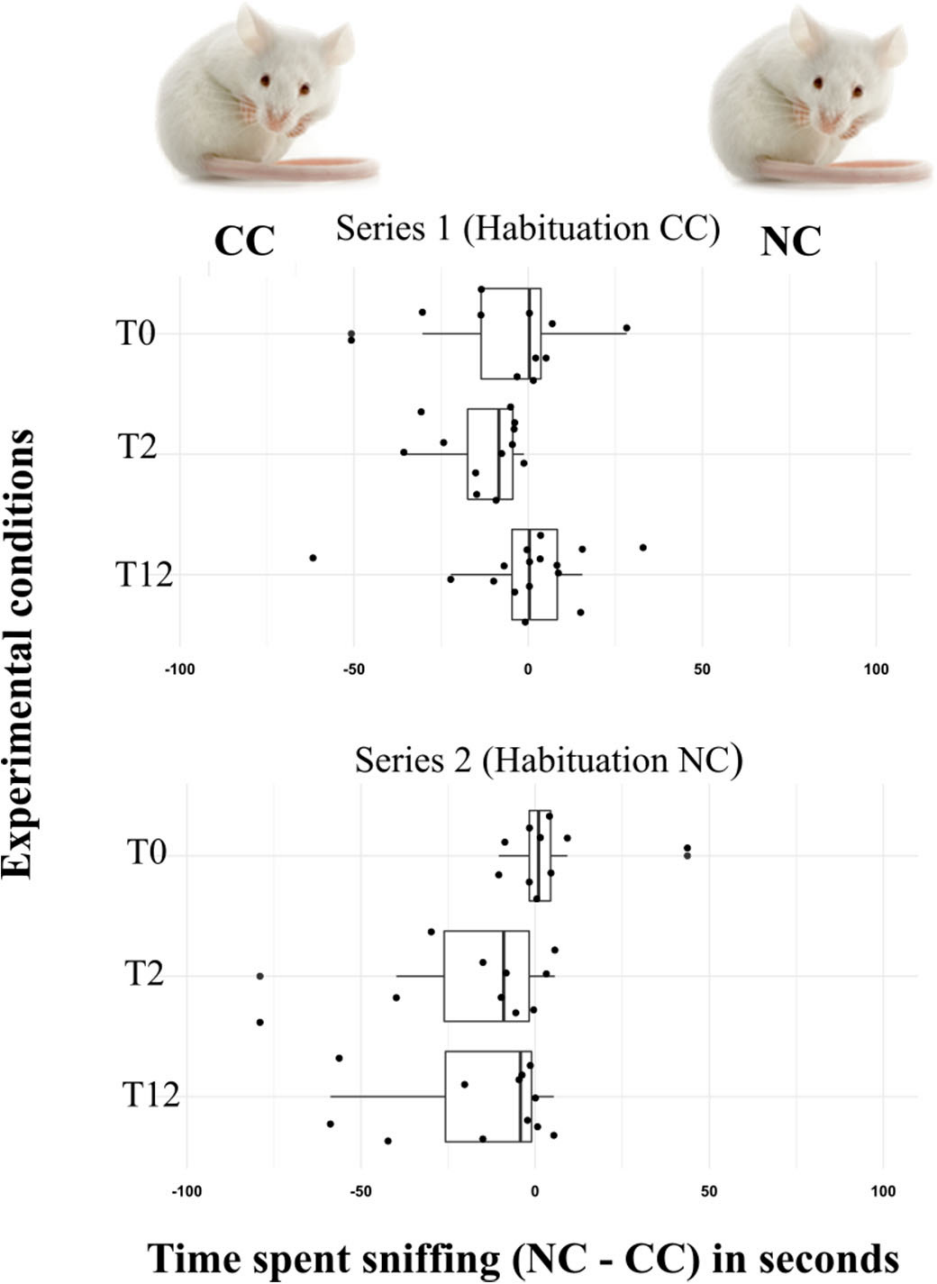
**Results of the generalisation tests.** Difference between time spent sniffing stimuli NC versus CC (in seconds) during the generalisation phase for series 1 (habituation to stimulus CC) and series 2 (habituation to stimulus NC) for each experimental condition (T0, T2 and T12). Number of males tested for each trial is: for series 1: *n*=11 for T0, *n*=12 for T2 and *n*=16 for T12; and for series 2: *n*=10 for T0, *n*=11 for T2 and *n*=12 for T12. A positive difference indicates that the mice spent more time sniffing a NC stimulus and a negative one indicates that the mice spent more time sniffing a CC stimulus. Boxplots include the median (vertical line) and the first and third quartiles, the whiskers represent the 95% confidence interval. Each dot represents an individual measurement.

Male mice were either habituated to a cancerous (CC), series 1, stimulus or a non-cancerous (NC) one, series 2, (see Fig. S3 for the habituation results), then presented with two other stimuli, a NC and a CC, during the generalisation phase. Based on our second preliminary test we did not expect the mice to discriminate between T0 type stimuli, and the results confirmed our predictions (series 1: *N*=11, V=26, *P*-value=0.58, power=65%, series 2: *N*=10, V=32, *P*-value=0.7, power=75%) Fig. 2.

Two weeks after the antibiotic was added to the mice diet (T2), we expected that if development of lung tumour was initiated in CC mice, the NC and CC stimuli should be perceived as different. Hence, in the first series of tests, after habituation to CC we expected either no discrimination (if no tumour developed yet) or higher investigation of the NC stimulus. However, unlike predicted, mice investigated more the CC stimulus (*N*=12, V=0, *P*-value=1, power=100%; Fig. 2) suggesting that the difference between the two CC stimuli was higher than between CC and NC. We also expected that after habituation to the NC stimulus (series 2), if tumour development started in T2, the CC stimulus would be investigated more intensively than the NC one. This time our results confirmed the prediction (*N*=11, V=6, *P*-value=0.007; Fig. 2), indicating that the two NC stimuli were more similar to each other than the NC and the CC stimuli. Considering the tests involving discrimination between the T12 stimuli, for which presence of cancer tumour was confirmed for CC and excluded for NC, we expected the CC and NC smells to differ and hence be discriminated. For series 1, after habituation to CC, we expected NC to be more investigated, nevertheless the mice did not show a significant discrimination between the stimuli (*N*=16, V=75, *P*-value=0.372, power=45%; Fig. 2). For series 2, after habituation to a NC stimulus, consistent with our prediction, mice discriminated significantly between NC and CC (*N*=12, V=29, *P*-value=0.010; Fig. 2), suggesting that NC and CC were more different than two NC stimuli.

### Male general behaviour

To address attractiveness of CC and NC odours for the males we compared the total duration of time spent investigating the habituation odour as well as latency to approach this stimulus. The males spent significantly more time sniffing the CC stimulus as compared to the NC stimulus, when presented during the habituation phase, irrespective of the experimental conditions (LMM, ‘health status’: *F*_1,12=_3.923, *P*-value=0.048), and the males latency to approach the CC stimulus was shorter than the latency to approach the NC stimulus (*F*_1,12=_5.129, *P*-value=0.024), suggesting attractiveness to the CC stimulus. Finally, duration of sniffing was the highest at T0 and the lowest at T12 (‘experimental conditions’: *F*_2,12=_5.051, *P*-value=0.006; post-hoc T0-T2: *P*=1; T0-T12: *P*=0.02; T2-T12: *P*=0.3) suggesting a reduction in attractiveness of all stimuli after 12 weeks of antibiotic diet (Table 1).

**Table 1.**
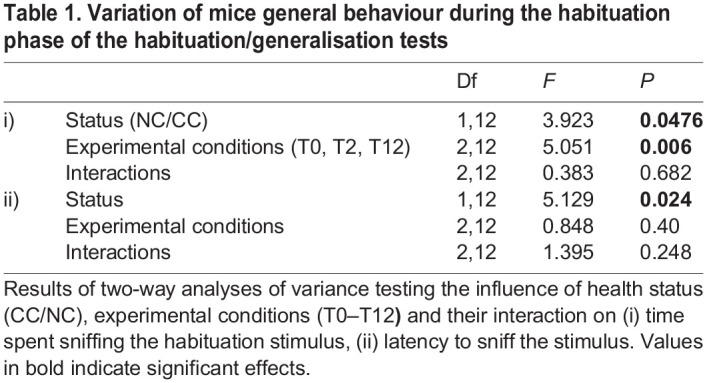
Variation of mice general behaviour during the habituation phase of the habituation/generalisation tests

### Variation of the VOC composition of the odour stimuli

Twenty-one compounds were identified in our biological samples after exclusion of molecules shared with the technical control. Three of the 21 compounds were excluded because of very low relative concentration (< 1%) and irregular detection in our samples (Table S1). We did not detect qualitative differences between CC and NC stimuli (Table S1).

**Fig. 3.**
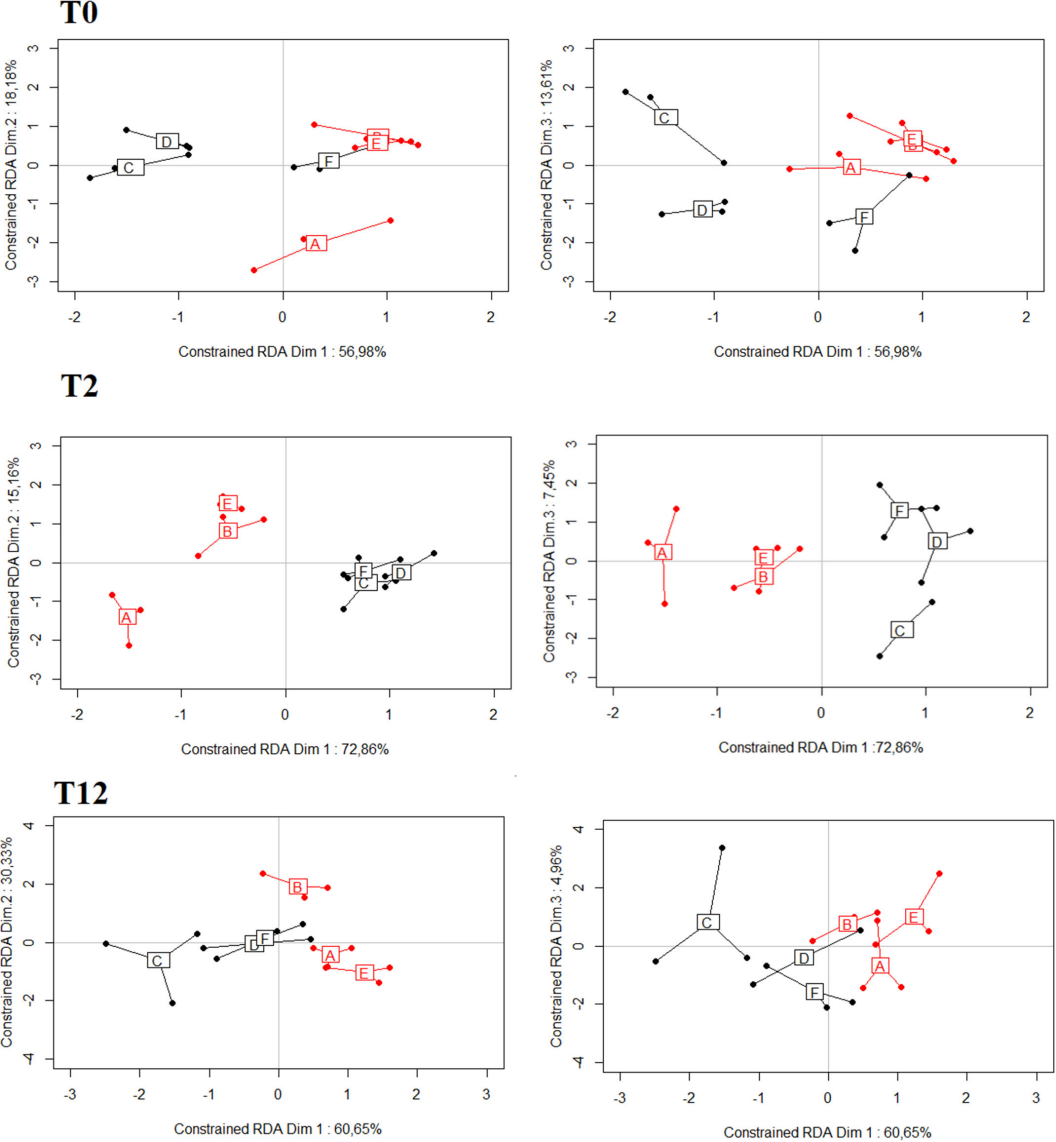
**Score plots of the redundancy analyses (RDAs) comparing variation of the relative concentrations of 18 VOCs with reference to health conditions (CC/NC) and the stimulus pools involved in the behavioural studies.** The left panels correspond to the projection of scores on the two first axes of the constrained RDA, and the right panels correspond to the projections on axes 1 and 3. A, B and E correspond to the three CC pools (in red) and C, D and F correspond to the three NC pools (in black).

We compared the relative concentration of the 18 molecules composing the odour bouquets of the behavioural stimuli. First, we tested the hypothesis of a higher heterogeneity among the CC stimuli as compared to the NC stimuli following the antibiotic treatment (experimental conditions T2, T12). A test of multivariate dispersion revealed that for both experimental conditions, variation among CC pools did not differ significantly from that among NC pools (T2: *F*^=^0.11, *P*=0.744; T12: *F*^=^0.001, *P*=0.971). Then, we assessed differences in the relative concentrations of the 18 VOC in the different pools with reference to the health status at each of the experimental conditions (T0, T2, T12). Results of the RDAs indicated that the variance among replicates of each pool accounted for 2.22 to 13.29% of the total variance, while the variance explained by health status and stimuli pools (constrained variance) accounted for 49.29% to 62.18% of the total variance. The permutation *F* test indicated that CC and NC pools were significantly different at the three experimental conditions (T0, T2, T12; *P*<0.001; Table 2), despite a significant heterogeneity between pools within status in T0 and T12 (Table 2, Fig. 3).

**Table 2.**
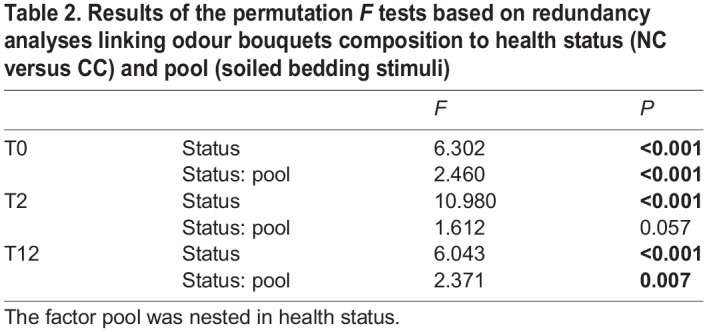
Results of the permutation *F* tests based on redundancy analyses linking odour bouquets composition to health status (NC versus CC) and pool (soiled bedding stimuli)

## Discussion

In nature, olfaction plays a key role in identifying the social status, age and health condition of potential sexual partners (Ali et al., 2015; Célérier et al., 2010; Desjardins et al., 1973; Heth et al., 2001; Hurst and Beynon, 2004; Latour and Ganem, 2017; Restrepo et al., 2006). The choice of a sexual partner is crucial for the chooser’s fitness. Olfactory mechanisms allow the detection of ill congeners, hence reducing the risk of contamination and of reproduction with congeners with lower fitness (Clayton, 1991; Ehman and Scott, 2001; Hurst, 2009; Kavaliers and Colwell, 1995; Poirotte et al., 2017; Thomas et al., 2005). Many studies have documented the ability of animals to discriminate against parasitized congeners (Ehman and Scott, 2002; Hurst, 2009; Kavaliers and Colwell, 1995; Kennedy et al., 1987; Poirotte et al., 2017). Concerning cancer, a pathology that can affect most metazoans (Leroi et al., 2003), consecutive modification of body odours has been largely described but very few studies have investigated the ability of individuals to detect a cancerous congener or examined whether and how this ability might impact intraspecific interactions. Cancer is relatively common in wild animals (Madsen et al., 2017). Our study reports that in a genetically engineered mouse model, lung cancer modifies the mouse odour signature and that these changes can be detected by wild-derived congeners at an early stage of the tumour development but does not influence female preference for the males' odours. Finally, although we identified quantitative differences in VOC composition of CC versus NC mice at the three stages of tumour development, these variations do not seem to parallel some of our behavioural results, suggesting that other odorant molecules, e.g. proteins, peptides, etc., present in the soiled bedding but not investigated in our study might be involved.

### Female mice preference

Avoidance of ill sexual partners by females is expected to influence their fitness directly by reducing their own risk of contagion in case of infectious diseases, and/or indirectly by increasing the viability and attractiveness of their offspring (Ehman and Scott, 2002; Kavaliers and Colwell, 1995). This avoidance can be based on information contained in the male odour signature (Clayton, 1991; Ehman and Scott, 2002; Hurst, 2009; Kavaliers et al., 2005; Penn and Potts, 1998). In this study, wild-derived females investigated to the same extent CC and NC stimuli, at the three experimental conditions. Both variation in the VOC relative concentrations and evidence that males could discriminate between CC and NC (see below) suggest that the females were probably able to discriminate between the two odour types, still discrimination did not induce a directional choice. Although absence of preference in T0 would indicate similar attractiveness of odours of males of the two lineages, absence of choice in T2 and T12 suggest that the odour of CC males was not repulsive, despite the fact that CC males carried full-blown cancer in T12. Consistent with our results, other studies reported that female mice may not be attracted to a healthy male when the alternative is a male carrying a cancerous melanoma (Kokocinska-kusiak et al., 2020).

This study results indicate that presence of a cancerous tumour may not influence female preference, unlike it does when the males carry parasites (Ehman and Scott, 2002; Kavaliers et al., 2005). Indeed, several studies have shown that chemical signals emitted by individuals carrying nematodes *Heligmosomoides polygyrus* (Ehman and Scott, 2001), protozoan *Eimeria vermiformis* (Kavaliers and Colwell, 1995) or murine louse *Polyplax serrata* (Kavaliers et al., 2003), were discriminated against by females, which usually chose an uninfected partner. However, unlike lung cancer or melanoma the parasites listed above could be transmitted to the females. Males at advanced stage of tumour development may not be able to defend a territory, protect a progeny, or keep a high social status, in natural conditions, and this should theoretically impact female preference (Hurst and Beynon, 2004). However, if in natural conditions cancer develops only at a late age, i.e. in non-reproductive animals, it should not impact reproduction and hence discrimination and avoidance of cancerous individuals may not be favoured by selection. Cancer in wild animals, in general, and more particularly among mice has been shown to be associated with aging. We know that rodents can develop cancer. Rats being the most tumour-prone rodent with a high tumour incidence at an early age but under laboratory conditions (Nakazawa et al., 2001). In other rodents such as laboratory gerbils (Vincent et al., 1979), laboratory mice (Frith et al., 1993; Greenacre, 2004; Ward, 1983), wild mice kept in laboratory conditions (Andervont and Dunn, 1962), pet chinchilla (Barbosa Lucena et al., 2012; Mans and Donnelly, 2013) or wild prairie dogs (Une et al., 1996), tumours were observed only in aging animals (Andervont and Dunn, 1962; Frith et al., 1993; Greenacre, 2004). Hence, cancer is probably rare in mice in the wild where longevity is estimated to be less than 1 year (Gerber et al., 2021), and the major causes of death in the wild are infectious diseases, predation or environmental perturbations (Vittecoq et al., 2013).

### Ability of males to detect cancerous congeners

The males were more attracted to CC than to NC stimuli, i.e. shorter latency and higher investigation duration of CC stimuli, suggesting that, unlike congeners carrying contagious illnesses, those carrying a cancer tumour may not be avoided. Our results also indicate that stimuli of mice that were subjected to a 12-week antibiotic treatment were less attractive than those of mice that did not receive an antibiotic treatment. Higher attractiveness of T0 than T12 odours may relate to the age difference of the two mouse types (Mucignat-Caretta et al., 2014) or to the impact of a long antibiotic diet. Further, wild-derived males were able to detect the tumour presence at an early stage of its development, i.e. two weeks after the antibiotic diet/lung oncogene induction. Surprisingly, our two series of experiments, differing by the health status of the stimulus presented during the habituation phase, yielded inconsistent results when the experiments involved T2 and T12 stimuli. Actually, when the habituation/generalisation test involved two CC stimuli (series 1), despite the fact that the CC donors were subjected to the same treatment, in T2, the two CC stimuli were treated as more distinct from each other than the CC and NC stimuli. It could be that the specific CC donors of the stimulus presented during the habituation phase did not develop a tumour in T2 and hence that the habituation stimulus was more similar to NC than to CC. Unfortunately, we cannot verify this hypothesis since the mice were euthanized only at T12.

We verified that at T12 all our CC mice stimuli donors developed lung cancer, and as expected none of the NC mice donors developed a tumour. Still, again, discrimination did not take place between CC and NC during series 1 (habituation to CC), suggesting that the CC stimulus presented during the habituation phase and the one presented during the generalization phase carried marked odour differences, equivalent to the differences between the habituation CC and the generalization NC. This intriguing result might be explained by a higher heterogeneity among CC stimuli, possibly linked to variations in the pattern of tumour development (Marjanovic et al., 2012), that would have induced greater heterogeneity in CC mouse chemical signature. The VOC are only part of the chemical signature of a mouse. Indeed the later also contains, e.g. proteins, peptides, sulphated steroids. Our results show differences in the VOC quantitative composition between CC and NC mice at all experimental conditions (T0 to T12), hence we could have expected discrimination between CC and NC in all our experiments. Further, the behavioural results suggest higher heterogeneity among the CC stimuli than among the NC ones, which are not reflected in the VOC composition of these stimuli. Research on mouse urine (Zhang et al., 2015) has shown that seven proteins could be potential biomarkers of lung cancer development, which may be also involved in our study, further analyses of the entire chemical signature of the CC and NC mice should help to clarify this issue.

### Concluding remarks

The results presented here show that wild mice can discriminate a cancerous congener from a healthy one at an early stage of cancer development. This suggests that the chemical signature of an individual bearing a cancer can change at a very early stage of the tumorigenesis. It also opens up applied medical perspectives on the early detection of cancer using odorant sources, when imaging techniques, the gold-standard in lung cancer detection, cannot detect the tumour.

Our results indicate that wild-derived females do not discriminate against cancerous males, and that males do not avoid smells of cancerous congeners, consistent with the fact that such behaviour may not evolve when cancer does not impact individual fitness. Cancer has been present since the onset of multicellularity (Aktipis and Nesse, 2013). Still, very few studies address ecological implications of oncogenic processes, which, however, seem to have a theoretically significant impact on animal evolutionary ecology and ecosystems functioning (Thomas et al., 2017; Vittecoq et al., 2013). Such processes should be investigated in organisms in which cancer develops at an early life stage (Aktipis and Nesse, 2013; Thomas et al., 2017; Vittecoq et al., 2013).

## MATERIALS AND METHODS

### Ethical clearance

All the precautions for animal welfare were followed and all behavioural protocols received ethics clearance from the Ethical Committee for Animal Experimentation (French Ministry of Higher Education, Research and Innovation) number E3417221 (for the behavioural experiments) and number 1645-22123 (for the transgenic mice).

### Animals

#### Odour donors

Scent stimuli were obtained from CCSP/EGFRTL transgenic mice bred at the IRCM (Montpellier Cancer Research Institute). This mouse model was generated by crossing the Tet-ON-EGFR^T790M/L858R^ transgenic mouse strain (hereafter EGFRTL) with the CCSP-rtTA strain (hereafter CCSP) carrying rtTA, an inverse tetracycline responsive element, under the control of a lung specific promoter. In CCSP/EGFRTL mice the EGFR gene contains both the oncogenic L858R mutation (causing EGFR constitutive activation in the absence of ligand EGF) and T790M gate-keeper mutation (conferring resistance to the first generation of EGFR inhibitors, i.e. gefitinib/erlotinib). This system allows the expression of EGFRTL specifically in the lung but only upon doxycycline (antibiotic) exposure, thus leading to the development of peripheral adenocarcinomas with bronchioloalveolar features in alveoli as well as papillary adenocarcinomas (Li et al., 2007; Mur et al., 2020). Mating crosses were kept in heterozygosity, thus resulting littermate could have any of the four possible genotypes: WT/WT, WT/EGFRTLtg, CCSPtg/WT and CCSPtg/EGFRTLtg. In our experimental setting we used WT/WT mice, lacking both CCSP and EGFRTL transgenes, and hence not able to develop a cancer upon doxycycline induction, NC mice, and CCSPtg/EGFRTLtg mice carrying both transgenes that develop a lung tumour upon doxycycline induction, CC mice.

The stimuli donors, NC and CC mice were all males, and they were maintained at the breeding facilities of the IRD (Institute of Research and Development) in Montpellier, from the age of 6–8 weeks. They were kept in groups of two to four mice in plastic transparent cages (26.8 cm W*21.5 cm L*14.1 cm H). Each cage contained the same quantity of sawdust, a cellulose square, hay, and a cardboard tunnel. During all the experimentation period the mice mass was controlled (euthanasia if loss of≥10% of mass). Tumour development was induced by feeding the animals with doxycycline-containing food pellets (1 mg/kg) from the age of 13 weeks until the age of 25 weeks. All mice were euthanised at the age of 25 weeks after transfer to the IRCM where their lungs were inspected. Both the non-cancerous status of all NC and the cancerous status of all CC mice aged 25 weeks were confirmed by necropsy and with H&E (Haematoxylin and Eosin) staining on FFPE (formalin-fixed paraffin embedded) lung sections.

#### Mice involved in the behaviour tests

Behavioural tests were carried out with wild-derived male and female *Mus musculus domesticus*, which were part of an outbred colony founded with mice trapped in southern Jutland (Denmark) in 2011. They were maintained in the animal facility of Montpellier University (RAM-CECEMA), under standardized conditions (photoperiod 12/12) and food and water were available *ad libitum*. They were housed in transparent plastic cages (20 cm L×35 cm W×14 cm H) containing bedding made of sawdust, hay, and a piece of cardboard box. A red plastic igloo equipped with a wheel was also present in each cage. After weaning at the age of 24 days the mice were kept in pairs (sister and brother) till the age of 8 weeks. Two weeks before the start of the experiments and during the entire experimental period, the males were kept isolated, while two unfamiliar females shared the same cage. Females involved in the choice tests were neither pregnant nor lactating but could have experienced pregnancy earlier in their life. Two days before being tested a mixture of male soiled bedding was added to the female cages to induce sexual receptivity (oestrus/proestrus) and synchronize their cycle, nevertheless, vaginal smears were also performed at the end of each behavioural test to ascertain females' receptivity. Preliminary tests were also performed and involved 11–14, 4–5-month-old mice from the same breeding colony, kept under the same experimental conditions as the test animals.

### Stimuli preparation

**Fig. 4.**
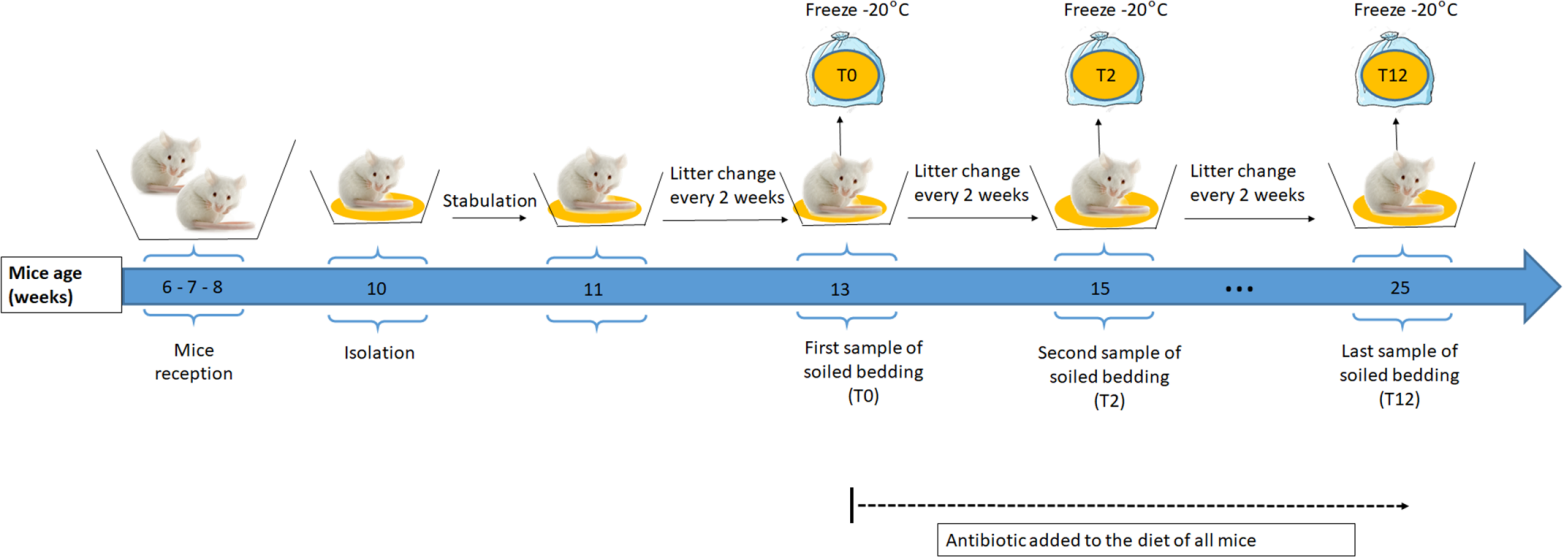
**Soiled bedding collection.** The same quantity of fresh bedding was provided to all mice (NC and CC genotypes, see text) every 2 weeks and the soiled bedding was collected at week 13 (T0), at week 15 (T2) and at week 25 (T12) and kept in a plastic bag at −20°C. The mice diet was supplemented with antibiotic from the age of 13 weeks and for 12 weeks. All mice were euthanized at the age of 25 weeks and screened for presence of cancer tumours.

The stimuli used for the behavioural tests were soiled bedding obtained from 17 males: eight CC and nine NC. Soiled bedding was a source of stimuli that contains a full range of odours emitted by the mouse. Each mouse was isolated at the age of 10 weeks in plastic transparent cages (26, 8 cm W×21, 5 cm L×14, 1 cm H) with 130 g sawdust and a cellulose square. The housing conditions of all odour donors were homogenized as much as possible. Each mouse was given the same quantity of bedding and food. Soiled bedding was collected every 2 weeks from each mouse and kept at −20°C. Clean bedding was also collected and kept in the same conditions. The first sample of soiled bedding was collected before feeding mice with doxycycline diet (hereafter T0), then 2 weeks (T2) and 12 weeks (T12) after starting the doxycycline diet (Fig. 4). Each scent stimulus was obtained by pooling soiled bedding of three to four mice to mitigate the effect of individual odours variation on the scent bouquet (details of pool composition for each test/experiment are given in Table 3).

**Table 3.**
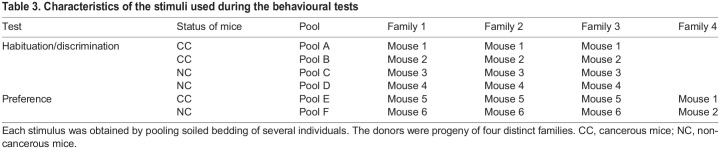
Characteristics of the stimuli used during the behavioural tests

#### Behavioural experiments

All the behavioural experiments were filmed to keep track of all tests (camera JVC Quad-Proof). We used the videos to record behaviour during the preference tests. Behaviour during the habituation/generalisation tests was live scored by an observer positioned 2 m away from the apparatus. The camera setup was placed before the start of any experiment. Data were recorded with the software Observer 5.0.31 (Noldus, 1991).

#### Experiment 1: female preference

Two-way choice tests were carried out using a Y-shaped maze connected to three boxes (15 cm L×15 cm W×10 cm H), a start box, and two peripheral boxes containing the stimuli. A female was placed in the start box for 2 min before the start of the experiment. During this time, two stimuli (4 g each) were placed at the far end of each of the peripheral boxes. The start box was connected to the Y maze just before the start of the experiment. Each test lasted 10 min starting when the mouse's two front paws and head were in the Y maze. If 5 min after the opening of the start box the mouse did not either enter the Y maze or visit one of the peripheral boxes, the test was not considered. The left and right position of the two stimuli was alternated between the tests to avoid biases due to lateralization.

A female was tested once a week and could have participated in up to three different tests each involving a choice between a pool of CC and a pool of NC collected during one of the three experimental conditions: T0, T2 or T12. The order of presentation of stimuli T0, T2 and T12 was randomized over the day and week. Out of 17 females, nine performed the three tests, the others, either because they were not in oestrus or because of long freezing during one of the tests, were involved either in only two tests (six) or a single test (two).

Preference for one of the two stimuli was inferred from comparisons of the relative time spent by a female in the box containing the NC stimulus versus the CC one.

#### Experiment 2: habituation/discrimination – habituation/generalisation

We used the habituation/generalisation test to assess differences in perception of similarities or of differences of CC versus NC odour sources (Fig. S1). The procedure started with a habituation phase during which a mouse was presented with a single stimulus until it got familiarized with it (reflected by the reduction of the time spent by the mouse investigating the stimulus over the course of the experiment). The second phase immediately followed the first, during which two new stimuli were presented to the mouse simultaneously (generalisation phase). During the generalisation phase, the mouse was expected to investigate less the stimulus most similar to the one presented during the habituation phase, unless the two stimuli were equally similar or different to the habituation stimulus (Todrank and Heth, 2003).

The apparatus was made of a Plexiglas transparent device comprising a starting box (15 cm L×15 cm W×10 cm H) connected to a test box (30 cm L×30 cm W×10 cm H) with a cylindrical tunnel (diameter 5 cm length 25 cm; Fig. S1). The mouse was introduced into the starting box, isolated from the rest of the apparatus by a sliding door, and left there for 1–2 min, while the habituation stimulus (4 g) was placed on the floor at the far end of the test box. The sliding door was then opened, and recording starts when the mouse two front paws were outside of the starting box. The habituation phase lasted 14 min. This phase was validated only if the mouse spent significantly more time investigating the stimulus during the first half than during the last half of the test. At the end of this phase, the mouse was isolated again in the starting box, and the habituation box was quickly replaced by a second test box containing two stimuli (4 g/stimulus let to thaw in the box) added 3 min before the end of the habituation phase and placed one on the left the other on the right side of the test box at 25 cm distance from each other. Not more than 30 s after the end of the habituation phase, the start box sliding door was opened again and the mouse was let to investigate the discrimination box for 7 min. To avoid bias due to laterality, the left and right position of the two stimuli was alternated between tests.

Considering the heterogeneity of tumour development (Marjanovic et al., 2012; Melo et al., 2013) and the potential impact of this heterogeneity on the mice odour signature, we expected greater variability between the CC stimuli than between the NC ones. To address this issue, we carried out two series of habituation/generalisation tests, during which the mice were habituated either to a CC stimulus, and then presented to a second CC and a NC stimulus (series 1; 20 mice) or habituated to a NC stimulus and then presented to a second NC stimulus and a CC one (series 2; 22 mice). Each mouse was involved in a maximum of three different tests during which it was presented with stimuli sampled at the three experimental conditions: T0, T2, T12. Two weeks after doxycycline feeding started (at T2), we expected that if development of lung tumour was initiated in CC, the NC and CC stimuli should be perceived as different. Conversely, if the tumour did not develop at T2 we expected that the NC and CC stimuli would not be perceived as different.

Habituation took place for nine mice at the three tests type, for 16 mice at only two of the tests and for 17 mice at only one of the tests. At least 1 week separated involvement of the same mouse in two tests, and the order of tests to which a mouse participated was randomized.

During this experiment we recorded: (1) the duration of investigation of each stimulus; (2) total time spent by a mouse in each box; and, (3) latency to investigate each stimulus.

##### Preliminary tests

These tests involved stimuli collected before starting the doxycycline diet (T0).

###### Preliminary 1: validation of the habituation/generalisation procedure

We performed a series of tests during which one of the two stimuli presented during the discrimination phase was the same as the one presented during the habituation phase. We expected that if discrimination occurred the more familiar stimulus (i.e. the one presented both during the habituation and the discrimination phase) would be less investigated by the mouse.

Eleven mice participated to the habituation phase, but only nine showed clear habituation and hence participated to the discrimination phase (see Fig. S2 for the habituation results). Mice were habituated either to a CC (four tests) or a NC (five tests) stimulus. The mice spent significantly more time investigating the less familiar stimulus during the discrimination phase (Mann–Wilcoxon test for matched samples, unilateral test, *N*=9, V=4, *P*=0.014) indicating that discrimination took place.

###### Preliminary 2: did the smells of CC and NC brothers differ?

To address this question, we used individual stimuli. Soiled bedding collected from three brothers two with a CC genotype and one with a NC genotype. Fourteen outbred mice were habituated to a CC stimulus (brother 1). The habituation phase was validated only for eight mice that participated in the generalization phase during which they were presented with a CC (brother 2) and a NC (brother 3) stimuli (see Fig. S2 for the habituation results). Time spent by the eight mice sniffing the two discrimination stimuli did not differ significantly (Wilcoxon test for matched samples, bilateral test, *N*=8, V=29, *P*=0.148, power=70%), indicating that the smell of two brothers with the same genotype (here CC) were not perceived as more similar to each other than odours of two littermates with a different genotype (CC/NC).

### Collection and identification of VOC present in the odour stimuli

**Fig. 5.**
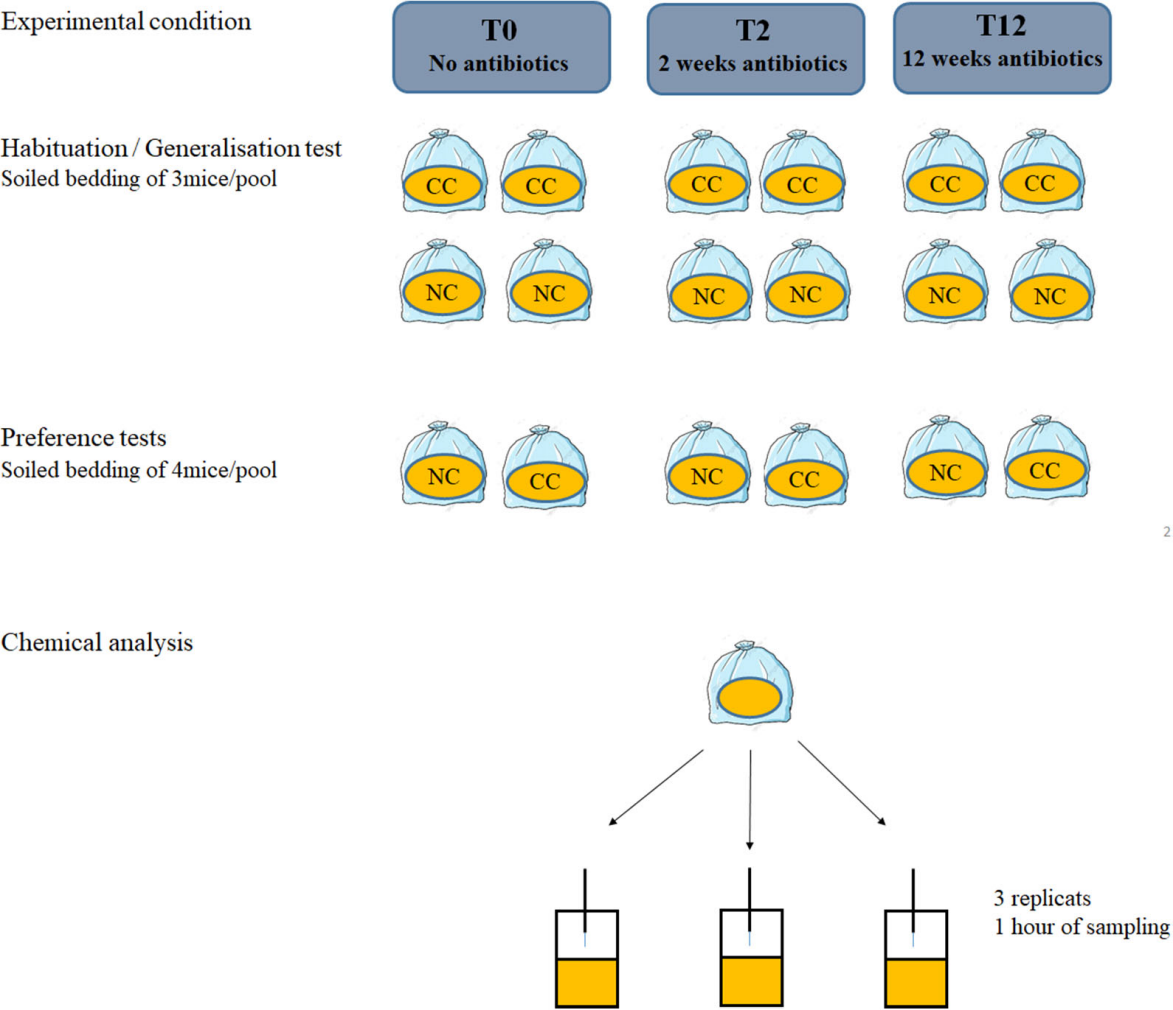
**Protocol for stimuli (soiled bedding) preparation and VOC extraction.** Each bag contained a stimulus made of a pool of soiled bedding obtained from four (pref. test) or three (habituation/generalisation) mice. We mixed 40 g soiled bedding/individual to obtain a 120 g bag for the habituation/generalisation tests and 20 g soiled bedding/individual to obtain 80 g of litter for the female tests. For the chemical analysis, we mixed 10 g soiled bedding/mouse.

We analysed the VOC composition of three samples (3 g each) per stimulus used during the behavioural tests (two during the habituation/generalisation and one during the preference test, per health status CC/NC). This approach was aimed to assess variation within and between the stimuli, which were pools made of soiled bedding of several individuals. In parallel, clean bedding, kept under the same conditions as the experimental stimuli, was also sampled and used as a technical control: VOC present both in the control and in the test stimuli were considered as non-informative (Fig. 5).

The VOC content of all samples was collected with a solid phase micro extraction fibre (SPME, 65 μm diameter PDMS-DVB composed of polydimethylsiloxane-divinylbenzene, Sigma-Aldrich, Bellefonte, PA, USA). An SPME fibre was exposed to a stimulus and analysed after desorption of the fibre content in a gas chromatographer coupled to a mass spectrometer (GC-MS). The stimuli and the control kept at −20°C were thawed and maintained on ice for the time of sampling. A 3 g sample was transferred into a 125 ml glass vial, which was then sealed. An SPME fibre was introduced into the vial after piercing its stopper with a needle. The position of the fibre in the vial was controlled so that the distance from the stimulus (∼2 cm) was similar between samplings. Following an equilibration time (3 min) in an oven maintained at 22°C, the SPME fibre was exposed to the stimulus for 1 h, before being introduced into the GC-MS injector (quadrupole mass spectrometer Shimadzu QP2010-SE (Shimadzu, Kyoto, Japan). The GC was equipped with an Optima 5-MS fused silica capillary column (30 m×0.25 mm×0.25 µm film thickness, Macherey-Nagel, Düren, Germany). Helium was used as carrier gas (1 ml min^−1^). The oven temperature was maintained at 40°C for 2 min, after which the temperature increased by 5°C every minute until it reached 175°C, and by 12°C min^−1^ until it reached 220°C. Injection of the SPME fibre in the GC for desorption was done while the injector was at 250°C. The chromatograms were analysed with the resident software (GCMS Solution, Shimadzu, Kyoto, Japan). We used the peak retention times (RT) and mass spectra to identify a compound. RT were then transformed into retention time indices (RI), using as a reference the retention time of a series of *n*-alkanes, that were injected in the same GC-MS (Alkanes standard solution, 04070, Sigma-Aldrich). Final identification of compounds was based on comparison with those in mass spectrum databases (NIST 2007, Wiley Registry Ninth) and RI available compounds databases (e.g. Adams, 2007, Pubchem, https://pubchem.ncbi.nlm.nih.gov/). Compounds that were present both in the technical controls (clean bedding) and in our studied samples (stimuli) were considered as potential pollutants and were excluded from the analyses. Compounds present as very small peaks and in a very small number of samples [three compounds: unknown compound 2; (x)-2,4,4-trimethyl-Pent-2-enal; 3,6,6-trimethyl-2-Norpinanone] were also excluded from the analysis. For the other compounds, we calculated their peak area on the total ion current chromatogram (TICC). Compounds present in the form of traces were given an arbitrary area value corresponding to 10% of the area of the smallest peak present in our dataset. Peak area for all compounds were then summed up per individual, and a relative surface area was calculated as the surface area of a given compound divided by the sum of all compounds for a given individual. Hereafter we refer to these values as relative concentrations.

### Statistics

All statistical analyses were performed with R version 3.4.4 in Rstudio (RStudio Team 2020 RStudio: Integrated Development for R. RStudio, PBC, Boston, MA, USA. URL http://www.rstudio.com). We used the following packages: vegan (Oksanen et al., 2020), lme4 (Bates and Maechler, 2009), ggplot2 (Wickham, 2014) and lmm (Schafer et al., 2020). Significance level was set at α=0.05. Normality and heteroscedasticity of the distribution of residuals were checked visually after plotting the model residuals, and data were transformed when it was necessary. G* power version 3.1.9.7 was used for power calculation (Faul et al., 2007).

#### Behavioural study

Wilcoxon signed ranks test (Siegel and Castellan, 1988) were used to compare paired variables: for the preference test, time spent by a given individual sniffing stimulus NC versus CC, and for the habituation/generalisation test, time spent, by a given individual, investigating the two stimuli presented during the generalisation phase. One-way tests were performed when our predictions were directional. Power tests (G*power) were performed to calculate the power of tests that failed to reject the null hypotheses.

LMM were applied to test the total time a mouse spent in contact with the two stimuli presented during the choice tests, with one fixed factor, experimental conditions (T0, T2, T12), and the mice identity as a random factor to control for the repeated use of some of the mice. To test variation of total duration of investigation of the habituation stimulus and latency to approach the habituation stimulus (i.e. did attraction or repulsion guide the mouse behaviour?), we also used the LMM procedure, with two fixed factors, experimental conditions (three modalities) and health status (touch modalities) and mice identity as a random factor.

#### VOC study

In order to compare VOC composition of CC and NC stimuli at each of the experimental conditions (T0, T2, T12) we used a multivariate approach [redundancy analysis, RDA, followed by a permutation *F* test (Hervé et al., 2018) R package vegan]. For this, relative proportions of VOC identified in the stimuli were first transformed using the Centred Log-Ratio transformation. As our data included zeroes, a constant value being one order of magnitude smaller than the smallest non-zero value (i.e. 0.001) was added to the entire data set. Then the data were autoscaled following the method proposed by Hervé et al. (2018). Three different pools of soiled bedding per health status were involved in the behavioural tests and this variable was included in the RDA as a fixed factor (three modalities) together with health status (two modalities).

To test whether heterogeneity in tumour development between CC mice may be reflected in their VOC composition, we compared multivariate dispersion within the odour bouquets among the CC and the NC pools using the function betadisper of the R package vegan (Euclidean distance based on clr-transformed and autoscaled VOC relative proportions). The test was performed separately for the two experimental conditions that followed antibiotic ingestion (T2, T12).

## Supplementary Material

Supplementary information
