# Supplementary material for: Odours of cancerous mouse congeners: detection and attractiveness
Source: Biol Open. 2022 Apr 29;11(4):bio059208. doi: 10.1242/bio.059208 (PMC9065363; doi:10.1242/bio.059208)
Supplement: Supplementary information [file biolopen-11-059208-s1.pdf]

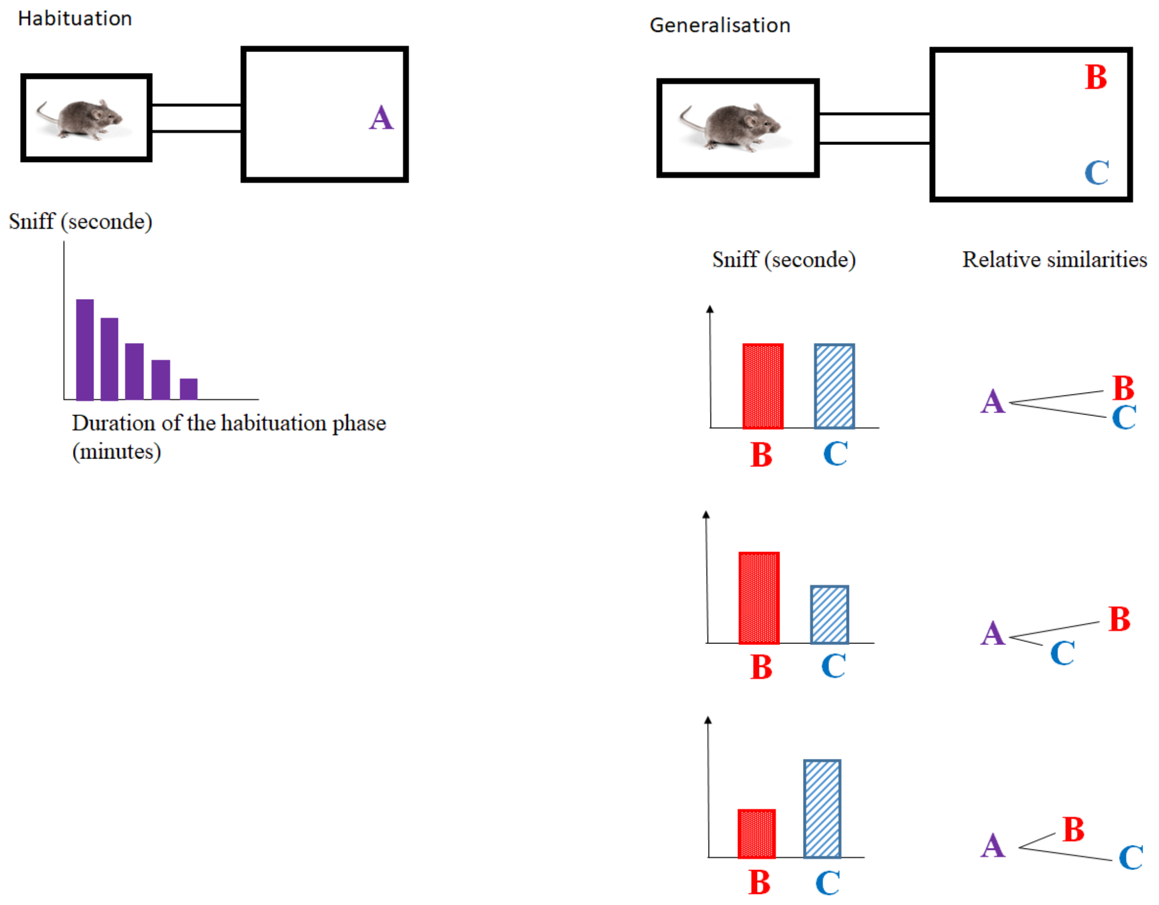

Fig. S1. A scheme describing the habituation / generalisation procedure and predictions. The generalisation phase follows the habituation phase only if the habituation phase is validated by a significant reduction of the time spent sniffing the habituation stimulus at the end of that phase. The generalisation phase allows evaluating relative similarities between the habituation stimulus and the two stimuli presented to the mouse during this phase.

Biology Open • Supplementary information

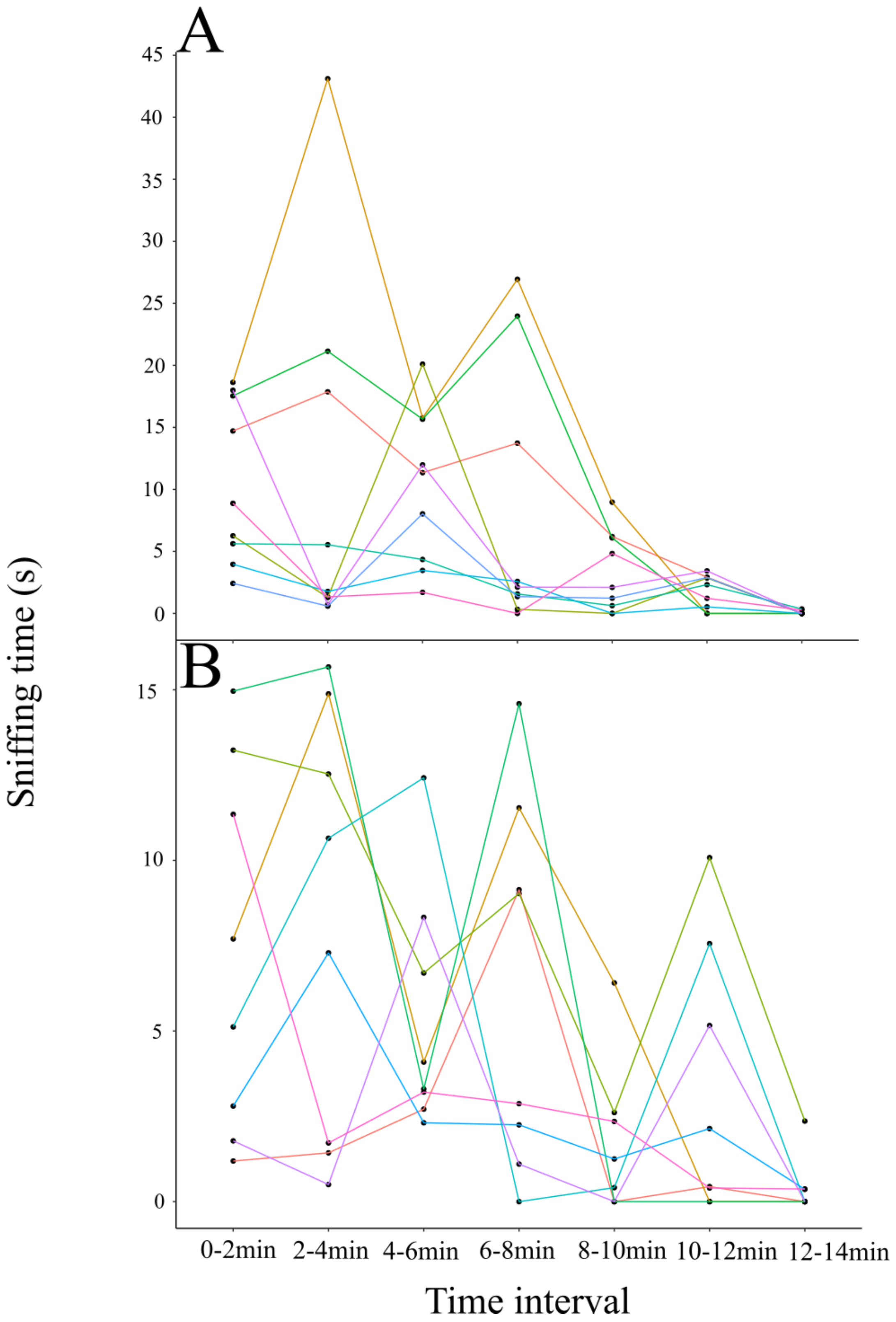

Fig. S2. Time spent by the mice sniffing the stimulus during the habituation phase for A) preliminary test 1 with n = 9 and B) preliminary test 2 with n = 8

Biology Open • Supplementary information

*Fig S3 : **Time spent by the mice sniffing the stimulus during the habituation phase for T0 A) series 1 with n =11 and B) series 2 with n = 10, for T2 C) series 1 with n = 12 and D) series 2 with n = 11 and for T12, for E) series 1 with n = 16 and F) series 2 with n = 12.***

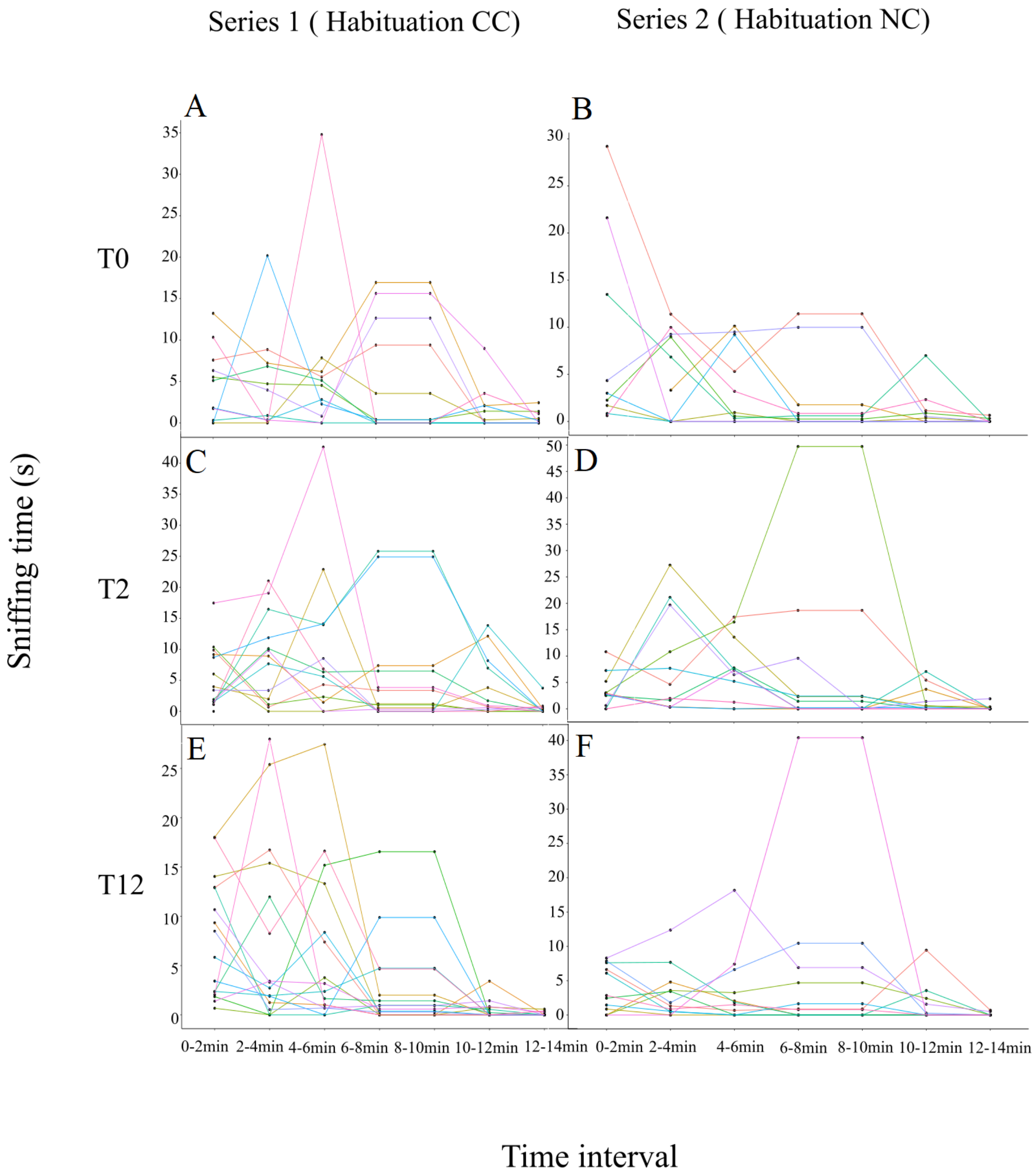

Fig. S3. Time spent by the mice sniffing the stimulus during the habituation phase for T0 A)series 1 with n =11 and B) series 2 with n = 10, for T2 C) series 1 with n = 12 and D)series 2 with n = 11 and for T12, for E) series 1 with n = 16 and F) series 2 with n = 12.

Biology Open • Supplementary information

Table S1. Identification of the 21 compounds, and their occurrence in the different stimuli used during the behavioural tests for NC (non-cancerous) and CC (cancerous) mice at the three experimental conditions (T0, T2 and T12). Retention indices (RI), retention times (RT).

Compound	RT	RI	NC			CC		
			T0	T2	T12	T0	T2	T12
Unknown compound 1	2.14	n.c	0	7/9	9/9	0	9/9	9/9
propan-2-ol	2.23	n.c	9/9	9/9	9/9	9/9	9/9	9/9
propanoic acid	4.31	707	9/9	9/9	9/9	9/9	9/9	9/9
3-methyl-butan-1-ol	4.89	733	3/9	8/9	9/9	8/9	9/9	9/9
butanoic acid	6.47	802	9/9	9/9	9/9	9/9	9/9	9/9
(x)-2-ethyl-hex-2-enal[TI]	6.68	809	0	9/9	9/9	9/9	9/9	9/9
Unknown compound 2 *	7.57	839	6/9	2/9	3/9	6/9	1/9	0/9
3-methylbutanoic acid	7.98	853	9/9	8/9	9/9	9/9	9/9	9/9
2-methylbutanoic acid	8.26	862	9/9	9/9	9/9	9/9	9/9	9/9
hexan-1-ol	8.45	869	9/9	9/9	9/9	9/9	9/9	9/9
(x)-2,4,4-trimethyl-pent-2-enal*[TI]	8.59	873	9/9	9/9	0	0	0	0
benzaldehyde	11.19	956	9/9	8/9	9/9	9/9	6/9	9/9
2,3-dehydro-exo-brevicomine	13.80	1039	9/9	9/9	9/9	9/9	9/9	9/9
3,4-dehydro-exo-brevicomine	14.06	1047	9/9	9/9	9/9	9/9	9/9	9/9
fenchone	15.36	1089	9/9	9/9	9/9	9/9	0	9/9
2-*sec*-butyl-4,5-dihydrothiazole	15.82	1103	9/9	9/9	9/9	9/9	9/9	9/9
nopinone	16.79	1135	9/9	9/9	8/9	9/9	8/9	9/9
camphor	17.06	1144	9/9	9/9	9/9	9/9	9/9	9/9
camphene hydrate	17.25	1150	9/9	9/9	9/9	9/9	9/9	9/9
(*E*)-3-pinocamphone	17.53	1160	9/9	9/9	9/9	9/9	1/9	9/9
3,6,6-trimethyl-2-norpinanone*[TI]	18.89	1205	8/9	7/9	6/9	0	0	9/9

** Compounds were removed from the subsequent statistical analyses because of their very low concentration (less than 1% of the mixture), and of uncertainty about their identification.*
n.c : not calculated
TI : attempt of identification; in some casesCC, the configuration could not be determinated.

Biology Open • Supplementary information
